# Upgrading Marine Oils from Cod (*Gadus morhua*) On-Board the Deep-Sea Vessels—From Waste to Value

**DOI:** 10.3390/foods12081659

**Published:** 2023-04-16

**Authors:** Line Skontorp Meidell, Ana Karina Carvajal, Turid Rustad, Eva Falch

**Affiliations:** 1 Department of Biotechnology and Food Science, Norwegian University of Science and Technology (NTNU), 7012 Trondheim, Norwayeva.falch@ntnu.no (E.F.); 2 Department of Fisheries and New Biomarine Industry, SINTEF Ocean, 7010 Trondheim, Norway; ana.k.carvajal@sintef.no

**Keywords:** cod, residual raw material, seafood processing, fish oil, quality, omega-3, food loss, food waste

## Abstract

Significant amounts of marine raw material are lost on-board the deep-sea vessels due to fast quality degradation. Optimal on-board handling and processing strategies can upgrade these resources from waste to food ingredients rich in nutrients such as omega-3 fatty acids. The objective of this study was to investigate the effect of raw material freshness and sorting on the quality, composition and yield of oil produced thermally from cod (*Gadus morhua*) residuals on-board a commercial trawler. Oil was produced from whole viscera fractions with liver or out-sorted livers right after a catch and after chilled storage for up to 6 days. The results showed that significantly higher oil yields could be obtained if the raw materials were stored for 1 day or longer. However, an undesired emulsion was formed when viscera were stored for 4 days. All oils were rich in health beneficial omega-3 fatty acids, but viscera oils had generally lower quality with higher levels of free fatty acids and oxidation products. However, out-sorting of the liver was not necessary to meet guidelines for high-quality fish oil. Both viscera and liver could be stored for up to 2 days at 4 °C prior to oil production and still meet quality criteria for food applications. These results demonstrate a large potential in upgrading currently wasted marine raw materials into high-quality food ingredients.

## 1. Introduction

Norway is the second largest fish producer in the world [1] and produced almost 3.8 million tons of seafood in 2021, including catches from fisheries and aquaculture [2]. Although the purpose of the seafood producers is to generate food, around 1.1 million tons (29%) of the obtained raw material was categorized as residual raw material. This raw material often gets lost early in the value chain or is underutilized to produce lower value products for feed, biogas or energy application [2]. The most significant losses are generated on-board the deep-sea vessels, where 70% of the residual raw material was discarded at sea in 2021 [2]. This part of the fishing fleet includes the largest vessels, mainly freezing trawlers and autoline vessels, which are out fishing for long periods far from shore. These vessels mainly produce headed and gutted (HG) whitefish with Atlantic cod (*Gadus morhua*), saithe (*Pollachius virens*) and haddock (*Melanogrammus aeglefinus*) as the most important species. The residual raw materials generated on-board are mainly viscera (including all inner fractions: liver, gonad, stomach and intestines) and heads. The viscera, and especially the liver, are rich in the health beneficial long-chain omega-3 polyunsaturated fatty acids (PUFA) eicosapentaenoic acid (EPA) and docosahexaenoic acid (DHA), which can be utilized to produce ingredients for human consumption [3]. However, these materials are exposed to fast quality degradation due to oxidation, enzymatic and microbial processes that start already a short time after the catch [4,5].

In order to produce high-quality ingredients from these raw materials, optimal handling and processing strategies are needed [6]. Thermal processing of marine oil from viscera or out-sorted livers can be an alternative on-board. In Norway, the production of marine oil from cod is traditionally based on out sorting of livers for the production of cod liver oil which is a labor-demanding procedure [7]. This industry is mainly based on the delivery of fresh livers from the coastal fleet with daily landings near the production facilities [8]. The deep-sea vessels face different challenges regarding utilization with large variations in available biomass due to differences in the amount of catch, by-catch and weather conditions [4]. This might imply the need for raw material storage prior to processing in periods on-board. However, it is a current knowledge gap on when to process the raw material as no published experiments have investigated the quality status and development in oil produced from cod residuals at an early stage on-board these large seagoing vessels. In addition, knowledge on the effect of an out-sorting of the liver compared to oil production from the whole viscera fraction is missing. Sorting of livers is usually done manually and is time-consuming and resource-intensive. Researchers have suggested that the use of computer vision can be a promising technology for the automatic sorting of cod livers on-board the deep-sea vessels [9]. However, the use of the whole viscera fraction would be less resource-demanding and increase the availability of raw material and omega-3 fatty acids on-board. 

To get a better understanding of these effects, this current study aimed to investigate the effect of raw material freshness and sorting on the quality, composition and yield of oil produced thermally from cod (*Gadus morhua*) residuals on-board a commercial trawler. The experiment was conducted on-board to enable oil production from whole viscera fractions and out-sorted livers immediately after a catch and during a storage period for up to 6 days at 4 °C. The yields of produced oil and emulsion were calculated, and the composition of the oil was analyzed by determination of fatty acid composition and distribution of lipid classes. To evaluate the quality of the oils, the content of free fatty acids and the amount of primary and secondary oxidation products were determined. The generated results provide valuable knowledge for decision makers on when and how to upgrade currently wasted residuals into high-quality ingredients for food applications on-board the deep-sea vessels.

## 2. Materials and Methods

### 2.1. Fishing Vessel, Gear and Study Area

The study was conducted on-board a commercial freezer trawler. The vessel had a length overall (LOA) of 66 m and 5170 horsepower (HP). The fish was captured with trawl using an Alfredo No. 5 trawl (polyethylene netting) with nominal mesh size of 133 mm. The trawl doors (Injector XF 9) had an area of 7.5 m^2^ and weight of 3000 kg with 90 m sweeps. The capture of the fish used for the experiment was conducted in the Norwegian Sea (70°31′ N/17°15′ E) in March 2021. The fish was captured in the same haul with a catch size of 26.3 metric tons, capture depth of 300 m and haul duration of 4.5 h. The main species captured in the haul were haddock (*Melanogrammus aeglefinus*), cod (*Gadus morhua*) and saithe (*Pollachius virens*).

### 2.2. Raw Material and Sampling Procedures On-Board 

Atlantic cod (*Gadus morhua*) (*n* = 60) with an average length of 67.4 ± 6.2 cm was used to collect liver and viscera for the experiment (Table 1). Each individual fish was randomly taken out from the production line after automatic stunning and manual bleeding by factory personnel. The total length was measured (from snout to tip of the tail) before manually gutting. The air temperature was 11 °C during the procedure. The sex of the fish was registered by visual investigation of the gonads (roe or milt). First, whole viscera fractions (including stomach, intestines, bile, liver and gonad) were carefully collected and weighed separately. The viscera were collected in plastic bags with viscera from 5 fish in each bag to a total of 5 bags. The same procedure was followed for the collection of out-sorted livers. The bags were marked to ensure the traceability of the data. The bags were closed tightly to minimize oxygen exposure and placed in the dark in a cold room at 4 °C until sampling at different storage days. The raw material was stored for up to 6 days with sampling at days 1, 2, 3, 4 and 6 before further processing and thermal treatment on-board. The bags collected for day 0 were not stored but processed within 2 h after the catch. The fresh, minced raw material was collected and frozen at −20 °C for later analysis of biochemical composition. The raw material was not grouped in relation to size or sex as the scope of the study was to investigate raw material available for commercial bulk oil production on-board. 

### 2.3. Oil Production by Thermal Treatment On-Board

At storage days 0 (within 2 h after the catch), 1, 2, 3, 4 and 6 the raw material from one bag of viscera (*n* = 5) and one bag of liver (*n* = 5) were minced separately on-board by using a kitchen grinder (10 mm holes). Minced raw material (approximately 40 g) was collected in 50 mL centrifuge tubes (*n* = 4 tubes) for each raw material. The minced mass was treated thermally to simulate industrial oil production and to ensure adequate inactivation of endogenous enzymes in the samples. The centrifuge tubes were closed with lids and placed in boiling water until the raw material reached a temperature of >90 °C. The tubes were covered with aluminum foil to minimize light exposure. A thermometer was used to ensure adequate heating. When the temperature reached 90 °C the tubes were incubated in the boiling water for 15 min to simulate a typical temperature and time combination used in the fish oil industry [10,11]. The tubes were centrifuged for 10 min at 2250× *g* to separate the mass. The samples were then stored dark at −20 °C during the cruise (24 days) and during transport (2 days) to the laboratory in Trondheim (Norway). After arrival, the samples were immediately frozen at −80 °C. Before further analyses, the frozen material in the centrifuge tubes from each storage day (*n* = 4 tubes) was separated into four fractions: oil, emulsion, stick water and sludge. The oil fractions from each storage day (*n* = 4 oil fractions) were collected with a spoon, mixed and flushed with nitrogen prior to lipid analysis. The stick water and sediments were separated with a scalpel. A thin emulsion layer was observed in some samples, and this was carefully collected with a spoon. All fractions were weighed and frozen at −80 °C. The yield of oil was calculated as the percentage of the total weight of the fractions obtained from each tube.

### 2.4. Chemicals 

The chemicals used for biochemical analyses were: chloroform, hexane, methanol, isooctane, acetic acid, pyridine, 2-thiobarbituric acid, sodium sulfite, trichloroacetic acid (TCA), p-anisidine (all from VWR International AS, Oslo, Norway), copper (II) acetate monohydrate (Thermo Fisher, Kadel, Germany), potassium iodide, sodium thiosulfate, 1,1,3,3-tetraethoxypropane (TEP), oleic acid and deuterated chloroform (99.96%, containing 1% tetramethylsilane) (all from Merck, Darmstadt, Germany). The chemicals were of analytical grade. 

### 2.5. Proximate Composition 

Dry matter and ash contents were determined gravimetrically by heating a sample of 2 g at 105 °C overnight and for 14 h at 550 °C [12]. The method of Bligh & Dyer [13] was used to extract the lipids, and the lipid content was determined gravimetrically. The nitrogen content was determined by the Kjeldahl method as described by Abel et al. [14]. The protein content was determined by calculating the nitrogen content with a factor for fish and meat of 6.25 [15]. The analyses were performed in duplicates. 

### 2.6. Fatty Acids Composition 

The fatty acid composition was determined by gas chromatography (GC) according to the procedure described by Dauksas et al. [16] and was performed in duplicates. The results are shown as % of total fatty acids. 

### 2.7. Free Fatty Acids (FFA)

The content of free fatty acids (FFA) was analyzed according to the method proposed by Bernárdez et al. [17] with the use of isooctane as solvent instead of cyclo-hexane. The analyses were performed in 4 parallels and expressed as % oleic acid in the oil. 

### 2.8. Lipid Classes

^1^H NMR spectroscopy was used to study the lipid classes in oil produced thermally from viscera and liver at storage days 0, 3 and 6. An amount of 120 mg of oil was transferred to 5 mm NMR tubes and dissolved in 0.6 mL deuterated chloroform (99.96%, containing 1% tetramethylsilane). ^1^H spectra were recorded on a Bruker Avance 600 MHz spectrometer (Bruker Biospin GmbH, Rheinstetten, Germany) with a cryo-probe operating at a ^1^H frequency of 600.23 MHz at ambient temperature (25 °C). ^1^H Pulse program was used with the following parameters: pulse program zg 30, number of scans 24, time domain 65K, acquisition time 3.0 s, relaxation delay 2 s, dummy scans 2 and spectral width 18.03 ppm. The ^1^H NMR was run quantitatively (recycling time > 3 × T1 for using 30° pulse) for peaks in the glycerol and methyl region, where the longest T1 is reported to be for methyl groups of fatty acids (with T1 of 1.5 s) [18]. The software Topspin 4.0.7 was used for data processing, and peaks were integrated manually. Determination of lipid classes was conducted by the integration of chemical shifts in the ^1^H NMR spectra based on the literature [18,19,20] and included the detection of signals appearing in the spectral regions of triacylglycerols (TAG), diacylglycerols (DAG), monoacylglycerols (MAG), cholesterol (Chol) and phospholipids (PL). Missing fatty acids (FA) represent fatty acids not detected as lipid classes and were calculated by relating the signals of total detected lipid classes to the signals in the region of “all fatty acids”. The determination of lipid classes and missing FA was based on the calculation of fatty acid equivalences out of total fatty acids (%).

### 2.9. Peroxide Value (PV)

The peroxide value (PV) was determined by iodometric titration according to ISO 3969 and the AOCS Official Method Cd 8b-90 b [21] and performed in 3–5 parallels. The results are shown as meq/kg oil.

### 2.10. p-Anisidine Value (AV) and Total Oxidation Value (TOTOX)

The p-anisidine value (AV) was determined according to AOCS Cd 18–90 [21] and was performed in triplicates. The total oxidation value (TOTOX) was calculated by the formula 2 × PV + AV.

### 2.11. Thiobarbituric Acid Reactive Substances (TBARS)

Thiobarbituric acid reactive substances (TBARS) were determined based on a method proposed by Ke and Woyewoda [22]. The procedure was followed as described by Cropotova et al. [23]. The results are expressed as μmol/g lipid and as an average of 4 parallels.

### 2.12. Statistics

Oil samples from each storage day (*n* = 4) were mixed prior to analysis. The number of analytical parallels is described for each specific method. The statistical analyses were conducted with SPSS software (IBM SPSS Statistics 27). One-way analysis of variance (ANOVA) with Tukey’s HSD test and *t*-test were performed to identify significant differences between oil samples and storage times, assuming normal distribution and equal variance. The significance level was set to *p* < 0.05 and results are expressed as mean values ± standard deviation (SD).

## 3. Results and Discussion

### 3.1. Raw Material Composition and Yield of Produced Oil

Analysis of the biochemical composition of fresh raw material showed that viscera contained 57.3 ± 8.5% water, 21.8 ± 2.9% lipids, 12.5 ± 0.3% protein and 1.0 ± 0.2% ash, while liver contained 30.7 ± 3.0% water, 64.7 ± 1.5% lipids, 6.9 ± 0.3% protein and 0.6 ± 0.1% ash. The composition of viscera was similar to what has been reported by Dauksas et al. [16], where viscera containing liver from Atlantic cod caught in the Trondheim fjord (Norway) contained 60.0% moisture, 21.0% lipids, 14.9% proteins and 4.4% ash. According to Aursand et al. [24], cod liver typically contains 60% lipids, 9% protein and 28% water, which is in accordance with the results in the present study. Guil-Guerrero et al. [25] and Jacobsen et al. [26] also reported a similar lipid content in Atlantic cod livers, which was 65.9% and 50–64%, respectively. The results were also in accordance with what was reported for Atlantic cod caught in the North Sea in the same season and with similar fish length as the present study (caught in February/March with a length of 60–70 cm) with an average lipid content of 64% in liver and 3% in viscera (without liver) [27]. However, there can be large variations in the biochemical composition of cod viscera and liver due to seasonal variations and factors such as diet, age and sex [27,28,29]. It has been reported that the lipid content of cod liver can vary as much as 5 to 78% [30]. 

**Figure 1:**
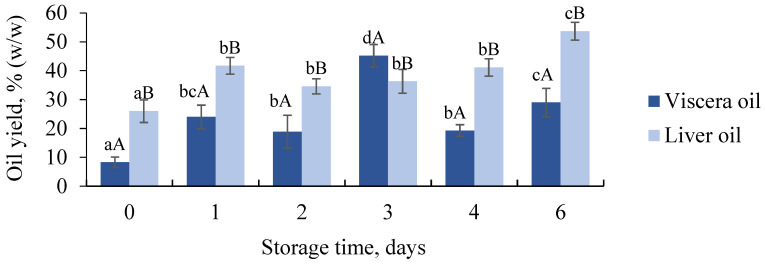
Oil yield (% *w*/*w*) obtained after thermal processing of viscera and liver at different storage times. Significant difference (*p* < 0.05) is shown as different letters between days for the same raw material (^a–d^) and between raw materials for the same day (^A–B^).

The yield of produced oil directly relates to the fisheries’ profits and knowledge about how the raw material freshness influences the oil yield is thus of interest to the industry. The lowest oil yields were obtained when the fresh raw material was used with yields of 8.2 ± 1.8% (*w*/*w*) for viscera and 26.0 ± 3.9% (*w*/*w*) for liver at day 0 (Figure 1). For both raw materials, the oil yields were significantly higher after storage for 1–4 or 6 days compared to fresh raw materials (day 0). For liver oil, the highest yield (53.7 ± 3.1% *w*/*w*) was produced on the last storage day (day 6). More time for proteolytic enzymes to hydrolyze proteins resulting in increased liberation of lipids from tissue and cells is most likely the reason for the increase. For viscera, the oil yield was highest at storage day 3 (45.2 ± 3.9% *w*/*w*) followed by lower yields at day 4 (19.3 ± 2.0% *w*/*w*) and 6 (29.0 ± 4.9% *w*/*w*). The high oil yield at day 3 was higher than the lipid content in the raw material (21.8 ± 2.9%). Some leakage of liquid due to gas formation in some of the tubes with viscera was observed during thermal treatment. This may have affected the results and was most likely caused by gas formation by gas-producing bacteria present in the intestines and stomach [4,31]. Although the minced viscera were mixed prior to sampling to ensure representative samples, insufficient collection of samples and small sample sizes (40 g per tube) with more liver mass might also have affected the yield. Biochemical differences in the viscera fractions might also be an explanation. The lower yields obtained at days 4 and 6 compared to day 3 were most likely caused by the formation of emulsion that was observed on the same days (8.2 ± 2.6% *w*/*w* at day 4 and 13.5 ± 2.0% *w*/*w* at day 6). Formation of emulsion typically results in lower oil yield and is undesired by the oil and protein-producing industry [32]. The emulsion was not observed on any other days or in liver samples. This was most likely due to the higher contents of phospholipids that are present in viscera, while the lipids in the liver are mainly present as triacylglycerols (TAG) or partial glycerides (mono- and diacylglycerols) [27]. Phospholipids tend to form emulsions [32] and increased liberation of phospholipids and changes in the proteins during storage, influencing their efficiency in forming emulsions might explain the formation after longer storage of the viscera.

The results indicate that oil should be produced from raw material stored for more than one day to maximize the oil yield and that viscera should be stored for a maximum of 3 days to avoid the formation of an emulsion. However, it must be kept in mind that differences between industry-scale and lab-scale processing might affect the results. Opheim et al. [33] compared laboratory-scale and industry-scale experiments on enzymatic hydrolysis of salmon residuals and suggested that differences in factors such as heating capacity and separating processes affected the composition of the produced ingredients. 

### 3.2. Fatty Acid Composition of Raw Material and Produced Oil

The dominating fatty acids in raw material and oil produced from viscera and liver were monounsaturated fatty acids (MUFA), followed by polyunsaturated fatty acids (PUFA) and saturated fatty acids (SFA). The viscera oils consisted of 50–55% MUFA, 26–28% PUFA and 19–22% SFA (Table 2). This is in accordance with results reported by Dauksas et al. [16], where oil produced by enzymatic hydrolysis of Atlantic cod viscera (including liver) consisted of 54–56% MUFA, 24% PUFA and 20–22% SFA. Falch et al. [27] reported that PUFA dominated in the viscera of Atlantic cod not containing liver. This indicates that major parts of MUFA in the viscera oils originate from the liver. The fatty acid composition of the liver oils contained 51–53% MUFA, 26–32% PUFA and 17–22% SFA (Table 2). Similar results have been reported for the liver of Atlantic cod in earlier studies [25,26,27,29], with MUFA, PUFA and SFA contents in the range of 37–54%, 29–41% and 13–27%, respectively. According to recent studies, intake of marine MUFA might have protective effects against different life-style related diseases [34]. However, the most well-documented health effects are associated with the intake of the marine long-chain PUFA EPA and DHA [3].

The average content of total omega-3 fatty acids accounted for 23.8% of all fatty acids in both raw materials (viscera and liver) (Table 2). The contents in oils produced during storage ranged from 22.4 to 23.5% for the viscera oils and from 20.3 to 24.2% for oils produced from out-sorted livers. Fish oil typically contains between 10 to 35% omega-3 fatty acids [11], indicating that the produced oils are good sources of these valuable fatty acids. European Pharmacopeia (Ph. Eur.) provides guidelines for the composition and quality of purified cod liver oil intended for medicinal products but is also commonly used in the pricing of crude fish oil for human consumption [24]. According to Ph. Eur. [35], cod liver oil should have a content of 7 to 16% EPA and 6 to 18% DHA. All oils were within these guidelines, with EPA and DHA contents of 7–10% and 8–10% in viscera oils and 7–10% and 6–9% in liver oils, respectively. Similar results have been reported for Atlantic cod livers by Jacobsen et al. [26], with EPA and DHA contents of 6–9% and 11–14%. The fatty acid composition in the liver of cod is affected by factors such as diet [36], seasonal variations [29,37] and reproductive status [27,29,38]. The fish used in the experiment was caught in the spawning period (January to April), which might explain the slightly lower DHA contents found in the present study.

Studies have reported reduced contents of PUFA and omega-3 fatty acids after longer storage of fish oil [39], minced residuals from saithe [40] and after storage of saithe and herring silage [40,41]. Only small differences in these contents were observed in the present study and no decreasing trend was observed after longer storage. Grinding and mincing can lead to faster oxidation compared to the storage of whole fractions [5]. Thus, storage of intact fractions on-board, as conducted in the present study, might be a desired solution to prevent parts of oxidation during storage. 

As expected, the fatty acid composition varied more for viscera oils than the liver oils and is most likely due to larger variations in the biochemical composition of viscera due to stomach content, gonad size and sex. The results confirm that such individual differences have a more important effect on the fatty acid composition than freshness and storage time. 

### 3.3. Lipid Classes in Produced Oil 

**Figure 2:**
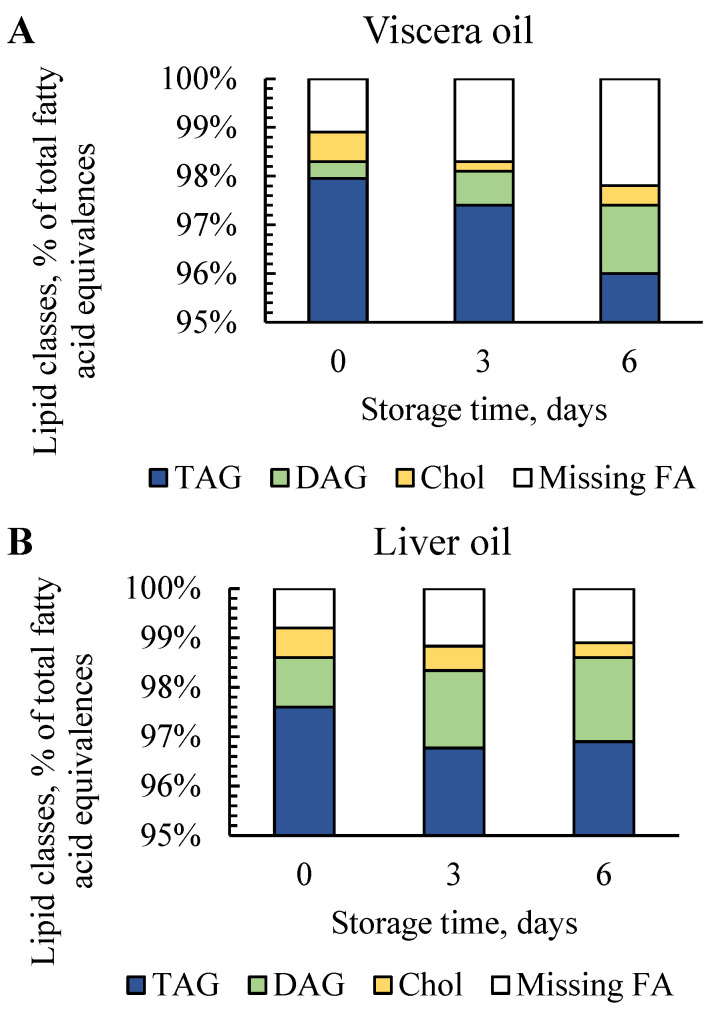
Lipid classes presented as fatty acid equivalences (% of total fatty acids) obtained from ^1^H NMR analysis in (**A**) viscera oil and (**B**) liver oil.

^I^H NMR spectra provided information about the lipid classes (% of total fatty acid equivalences) based on regions found in the literature [18,19,20]. Lipid classes were determined in oils produced at days 0, 3 and 6, where signals appeared in the region of triacylglycerols (TAG), diacylglycerols (DAG) and cholesterol (Chol) (Figure 2). Signals in the spectral regions of monoacylglycerols (MAG) and phospholipids (PL) were below the detection limit. Missing fatty acids (FA) were calculated based on the signals in the region of “all fatty acids” and includes fatty acids that were not detected as TAG, DAG or Chol. This may include lipid classes such as free fatty acids (FFA) that cannot be detected in ^1^H NMR spectra (FFA content was determined and is discussed in Section 3.4).

Viscera oil produced from fresh raw material (day 0) contained 98.0% TAG, 0.4% DAG and 0.6% Chol. Liver oil made of fresh liver contained 97.6% TAG, 1.0% DAG and 0.6% Chol. This is in accordance with a study by Dauksas et al. [16] that reported a TAG content of 96–98% in oil produced by enzymatic hydrolysis of cod viscera containing liver. The results are also in accordance with a study by Falch et al. [27] that reported a TAG content of 95–96% in lipids extracted from Atlantic cod livers frozen right after the catch. The higher amount of DAG in liver oils compared to viscera oils on all days (days 0, 3 and 6) is most likely due to the higher concentration of lipases in cod liver compared to viscera [42,43], resulting in lipolysis of TAG into DAG and FFA. 

Changes in lipid classes during storage can be caused by factors such as lipolysis by lipases, the liberation of lipids from tissue and cells during storage, increased availability of substrates for the lipases and oxidation. For viscera oil, the DAG contents increased from 0.4% at day 0 to 0.7 and 1.4% at days 3 and 6. DAG in liver oils increased from 1.0% at day 0 to 1.6 and 1.7% after 3 and 6 days of storage (Figure 2). This is in accordance with a study by Falch et al. [44] who reported that the DAG content in lipids extracted from Atlantic cod roe increased from 0.2% in fresh roe to 1.9% after 7 days of raw material storage at 4 °C. The increase in DAG was likely caused by lipolytic activity during storage. It could be assumed that the missing FA, where FFA can be located, were higher in liver oils due to the higher DAG contents in these oils compared to viscera, but the opposite was observed. Factors that might explain the results could be a higher amount of FFA already present in the stomach content of the viscera or diffusion of some FFA into the rest fraction after centrifugation. However, the amount of FFA cannot be directly correlated to the amount of missing FA. Still, the indication of lower FFA contents due to lower missing FA in oil produced from liver compared to viscera was in accordance with results obtained from the FFA analysis discussed in Section 3.4. 

Longer storage led to slightly lower contents of Chol in both viscera oil (0.6% at day 0, 0.2 and 0.4% at days 3 and 6) and liver oil (0.6% at day 0, 0.5 and 0.3% at days 3 and 6) (Figure 2). This has been observed in previous studies during the storage of whole fish and residuals from cod and saithe [40,44,45], where the decrease was explained by the increased content of cholesteryl esters formed by Chol and FFA. Overall, the results indicate that the composition of lipid classes was affected by both raw material freshness and sorting. 

### 3.4. Free Fatty Acids (FFA) in Produced Oil

**Figure 3:**
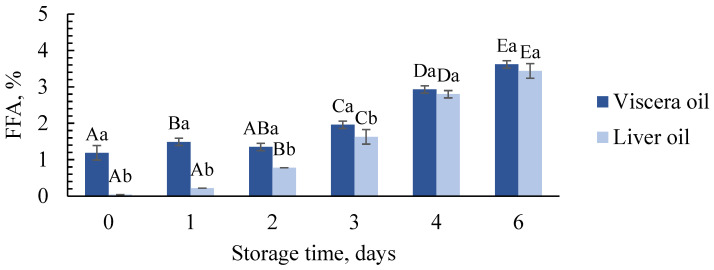
FFA content (as % oleic acid) in oil produced from viscera and liver at different storage days. Significant difference (*p* < 0.05) is shown as different letters between days for the same raw material (^A–E^) and between raw materials for the same day (^a–b^).

The amount of free fatty acids (FFA) is an important quality indicator for fish oil and reflects the lipolytic action of lipases present in the raw material [11,46]. The FFA content in viscera oil was, as expected, lowest in oil produced from fresh raw material (1.2 ± 0.2%) and increased significantly after 3, 4 and 6 days of storage compared to days 0–2 (Figure 3). The content was highest at day 6 (3.6 ± 0.1). For liver oil, no FFA was observed in oil produced at day 0. The content increased significantly between each storage day from day 1 and was highest on the last day (3.4 ± 0.2% at day 6). More time for the lipolysis to occur is likely the most important factor for the increase after longer storage. The liberation of lipases and lipids from the tissue during storage due to proteolytic breakdown may also bring the lipases closer to their substrate. Increased microbial activity after longer storage may also increase the FFA content due to lipolytic bacteria [47,48]. Sorting affected the quality, especially the first days of storage (days 0–3), where the FFA contents were significantly higher in viscera oil compared to liver oil. This might be caused by FFA already present in the stomach or intestines in the viscera as the diet affects the lipid composition [28]. High FFA contents in the raw material of cod viscera compared to liver have been demonstrated in earlier studies [27].

The content of FFA in crude fish oil intended for food applications is usually in the range of 1–7% [49]. This indicates that oil produced from both viscera and liver was of high quality and acceptable for food applications even after 6 days of storage of the raw material. The Global Organization for EPA and DHA Omega-3s (GOED) recommends an FFA content of ≤1.5% FFA (acid value 3 mg KOH/g) for refined fish oil [50]. Although the oils produced in the present study are unrefined, both viscera and liver oil were below this limit until day 3 (days 0–2). Further refinement steps such as neutralization with the addition of caustic soda or citric acid can be used to remove parts of the FFA in the oil if longer storage is necessary [11]. However, extensive refinement is resource-demanding and a lower FFA content will be more profitable for the fishing vessel as the FFA content is used in the pricing of crude fish oil [24]. 

### 3.5. Oxidative Status in Produced Oil 

The oxidative status of fish oil is an important quality parameter and quality recommendations are usually based on PV and AV analyses and calculation of TOTOX. PV indicates the amount of primary oxidation products that are further degraded to secondary oxidation products that can be measured by methods such as AV and TBARS. 

For viscera oil, the results of PV and AV were between 1.5 ± 0.5 and 12.0 ± 1.0 meq/kg oil and 1.1 ± 0.2 and 8.5 ± 0.3 (Table 3). Liver oil had PV values between 0.1 ± 0.0 and 2.5 ± 0.5 meq/kg oil and AV between 0.8 ± 0.1 and 5.4 ± 0.1. The TOTOX value in oil made of fresh viscera was 8.1 and increased to 9.5 at day 1 followed by an unexpected decrease at days 2 and 3 (5.6 and 5.4). The TOTOX in oil produced in the last days (days 4 and 6) were more than three times higher (28.6 and 25.8) than at day 0. The TOTOX value for oil produced from fresh liver was the lowest of all produced oils (2.3 at day 0). The value increased after a longer storage and was highest at days 3 and 4 (values of 8.7 and 7.8) and decreased to 4.7 at day 6. The unexpected decreases were caused by low PV values. Decreases in PV and AV after longer storage were also seen in a study on crude oil extracted from herring residuals stored at 2 °C [51]. This was explained by the instability of oxidation products and the possibility for complex formation with other compounds such as proteins, peptides or free amino acids. An unexpected decrease in secondary oxidation products (measured by TBARS) was also observed throughout the storage period in a study on pollock residuals stored at 6 and 15 °C [52].

GOED and Ph. Eur. have developed quality guidelines for refined fish oil intended for human consumption with the following recommendations: PV ≤ 5 meq/kg and ≤10 meq/kg oil, AV ≤ 20 and ≤30 and TOTOX ≤ 26 (GOED) [35,50]. The PV in oils produced in the present study were all under five, except oil made from viscera at days 4 and 6 (12.0 ± 1.0 and 8.6 ± 0.4). For AV, all oils were within the recommended levels in the guidelines. The TOTOX values were also within the requirements except for viscera oil at day 4 (28.6) and right below the limit at day 6 (25.8). This indicates that high-quality oil was produced from both viscera and liver during the first 3 days of storage. Sorting affected the quality, and out-sorted livers could be stored longer as high-quality oil was produced even after 6 days of storage. 

Due to the complexity of secondary oxidation products formed during oxidation, it can be valuable to use several oxidation methods. There are no quality guidelines for TBARS in fish oil, but an overview provided by Aursand et al. [24] showed that TBARS in marine oils for food supplements ranged between 0.1 and 3.7 μmol/g lipid. All TBARS were within this range, indicating that the oils were of high quality. The lowest values were observed in oil made of fresh raw materials, and the highest values were found in oils made on the last storage days. In general, the results showed slightly higher TBARS values in viscera oil compared to liver oil and the overall results supported the AV and TOTOX results.

## 4. Conclusions

The results indicate that storage of viscera and liver can be considered to increase the yield of produced oil. Significantly higher yields were obtained when the raw materials were stored for 1 day or longer at 4 °C prior to processing. However, storage of viscera for longer than 3 days would not be recommended as undesired emulsion was formed, resulting in decreased oil yields. The fatty acid composition did not seem to be affected by the raw material freshness, and oil produced from both raw materials was rich in the health beneficial omega-3 fatty acids EPA and DHA. Oil produced a short time after the catch led to higher TAG contents and lower FFA contents compared to oil produced from stored raw materials. Use of the whole viscera fraction resulted in oil with significantly higher FFA contents compared to liver oils on the first days of storage (days 0–3), but high-quality oil could be produced from both raw materials even after 2 days of storage according to quality guidelines. The amount of oxidation products was affected by both raw material freshness and sorting. The use of the whole viscera fraction led to higher oxidation, measured by TOTOX, in 4 of the 6 storage days. Based on quality recommendations for PV, AV and TOTOX, high-quality oil acceptable for food applications was produced even after 3 days of storage of the viscera and during the whole storage period for the liver. 

Overall, the results indicate that the raw materials should be processed a short time after the catch to optimize the oil quality, especially when utilizing the viscera. However, storage of the raw material for 1 to 2 days at 4 °C can be considered to meet both process and quality parameters important for the industry. Although out-sorting of the liver led to oil with higher quality, utilization of the whole viscera fraction should be considered as oil of high quality could be obtained, and this will increase the availability of raw material on-board and be a less resource-demanding procedure. However, potentially high bacteria contents in the viscera could be a limiting factor that should be investigated in future studies. This would be especially important considering the utilization of the remaining protein fraction after oil extraction. Overall, the obtained results can be used as a knowledge base for decision makers on when to process and how to upgrade highly degradable raw materials into high-quality food ingredients on-board the deep-sea vessels. 

## Figures and Tables

**Table 1:** Experimental data (fish length, raw material weight and sex) for raw material collected for each storage day.

Raw Material Fraction	Storage Day	Fish Length, cm		Viscera Weight, g		Sex	Raw Material Fraction	Storage Day	Fish Length, cm		Liver Weight, g		Sex
Viscera	0	65		656		♀	Liver	0	72		92		♂
Viscera	0	77		534		♂	Liver	0	75		120		♂
Viscera	0	77		544		♂	Liver	0	74		102		♂
Viscera	0	75		392		♂	Liver	0	68		90		♂
Viscera	0	62		244		♂	Liver	0	76		104		♀
Viscera	1	81		384		♂	Liver	1	74		180		♂
Viscera	1	66		402		♂	Liver	1	71		124		♂
Viscera	1	65		230		♀	Liver	1	68		100		♀
Viscera	1	59		162		♀	Liver	1	68		168		♂
Viscera	1	68		270		♀	Liver	1	68		62		♂
Viscera	2	65		378		♀	Liver	2	60		52		♂
Viscera	2	60		258		♂	Liver	2	71		134		♀
Viscera	2	75		788		♂	Liver	2	67		82		♂
Viscera	2	71		558		♂	Liver	2	73		122		♀
Viscera	2	65		450		♂	Liver	2	59		76		♀
Viscera	3	73		580		♂	Liver	3	67		60		♂
Viscera	3	62		178		♂	Liver	3	56		128		♂
Viscera	3	73		662		♂	Liver	3	55		36		♀
Viscera	3	61		218		♀	Liver	3	56		62		♀
Viscera	3	72		594		♂	Liver	3	56		46		♀
Viscera	4	71		302		♂	Liver	4	76		144		♀
Viscera	4	55		210		♀	Liver	4	68		154		♀
Viscera	4	71		294		♀	Liver	4	65		90		♀
Viscera	4	61		256		♀	Liver	4	64		112		♀
Viscera	4	61		112		♀	Liver	4	72		106		♂
Viscera	6	69		342		♂	Liver	6	74		124		♂
Viscera	6	64		622		♀	Liver	6	68		176		♀
Viscera	6	68		290		♂	Liver	6	69		152		♀
Viscera	6	66		408		♂	Liver	6	68		270		♂
Viscera	6	61		282		♂	Liver	6	67		184		♀
*Viscera*	* Average		*67 ± 6*		*383 ± 175*		*Liver*	* Average		*68 ± 6*		*115 ± 50*	

* Average ± standard deviation (SD) of the samples (*n* = 30).

**Table 2:** Fatty acid composition (% of total fatty acids, mean ± SD) in raw material of viscera and liver and in oil produced after different days of storage.

**Viscera**							
	**Raw Material**	**Oil** **Day 0**	**Oil** **Day 1**	**Oil** **Day 2**	**Oil** **Day 3**	**Oil** **Day 4**	**Oil** **Day 6**
EPA	9.2 ± 0.0 ^Aa^	8.9 ± 0.2 ^Abc^	8.9 ± 0.0 ^Ab^	9.0 ± 0.0 ^Aab^	9.5 ± 0.0 ^Ad^	7.4 ± 0.0 ^Ae^	8.6 ± 0.0 ^Ac^
DHA	9.1 ± 0.1 ^Aa^	8.5 ± 0.2 ^Ab^	8.6 ± 0.0 ^Ab^	7.7 ± 0.1 ^Ac^	7.8 ± 0.0 ^Ac^	10.1 ± 0.0 ^Ad^	9.2 ± 0.0 ^Aa^
SFA	22.4 ± 0.1 ^Aa^	20.9 ± 0.0 ^Ab^	20.9 ± 0.1 ^Ab^	19.1 ± 0.2 ^Ac^	19.4 ± 0.1 ^Ac^	22.1 ± 0.1 ^Aa^	20.1 ± 0.1 ^Ad^
MUFA	49.5 ± 0.1 ^Aa^	52.1 ± 0.4 ^Ab^	51.8 ± 0.1 ^Ab^	54.7 ± 0.3 ^Ac^	53.6 ± 0.0 ^Ad^	50.6 ± 0.0 ^Ae^	52.0 ± 0.0 ^Ab^
PUFA	28.0 ± 0.0 ^Aa^	27.0 ± 0.5 ^Ab^	27.3 ± 0.0 ^Abc^	26.3 ± 0.0 ^Ad^	27.0 ± 0.0 ^Ab^	27.3 ± 0.1 ^Abc^	27.9 ± 0.1 ^Aac^
Omega3	23.8 ± 0.0 ^Aa^	23.0 ± 0.4 ^Abc^	23.1 ± 0.1 ^Ab^	22.4 ± 0.0 ^Ac^	23.3 ± 0.1 ^Aab^	23.2 ± 0.0 ^Aab^	23.5 ± 0.1 ^Aab^
**Liver**							
	**Raw material**	**Oil** **Day 0**	**Oil** **Day 1**	**Oil** **Day 2**	**Oil** **Day 3**	**Oil** **Day 4**	**Oil** **Day 6**
EPA	8.5 ± 0.1 ^Ba^	8.3 ± 0.0 ^Bb^	9.5 ± 0.1 ^Bc^	7.4 ± 0.0 ^Bd^	6.8 ± 0.0 ^Be^	8.5 ± 0.0 ^Ba^	8.0 ± 0.0 ^Bf^
DHA	9.3 ± 0.2 ^Ba^	8.7 ± 0.1 ^Ab^	9.1 ± 0.1 ^Bac^	8.8 ± 0.0 ^Bbc^	6.4 ± 0.0 ^Bd^	9.3 ± 0.0 ^Ba^	8.4 ± 0.1 ^Bb^
SFA	21.5 ± 0.3 ^Bab^	21.5 ± 0.3 ^Bab^	19.2 ± 0.1 ^Bc^	22.2 ± 0.3 ^Ba^	17.0 ± 0.0 ^Bd^	20.7 ± 0.0 ^Bb^	21.4 ± 0.2 ^Bab^
MUFA	50.8 ± 0.3 ^Ba^	51.9 ± 0.4 ^Abc^	52.5 ± 0.1 ^Bc^	51.4 ± 0.4 ^Bab^	51.5 ± 0.0 ^Babc^	51.9 ± 0.0 ^Bbc^	52.5 ± 0.2 ^Ac^
PUFA	27.7 ± 0.0 ^Ba^	26.7 ± 0.1 ^Bb^	28.3 ± 0.2 ^Bc^	26.4 ± 0.1 ^Abd^	31.5 ± 0.0 ^Be^	27.4 ± 0.0 ^Aa^	26.1 ± 0.0 ^Bd^
Omega3	23.8 ± 0.3 ^Aa^	22.6 ± 0.0 ^Bb^	24.2 ± 0.2 ^Ba^	22.1 ± 0.0 ^Abc^	20.3 ± 0.0 ^Bd^	23.2 ± 0.0 ^Ae^	21.9 ± 0.1 ^Bc^

Significant difference (*p* < 0.05) is shown with different letters between viscera and liver within each fatty acid group (^A–B^) and between different samples (raw material or oil produced at different storage days) within each fatty acid group (^a–f^).

**Table 3:** PV (meg/kg oil), AV, TOTOX and TBARS (μmol/g lipid) values in oil produced from viscera and liver at different storage times (mean ± SD).

	**PV (meq/kg Oil)**		**AV**	
**Day**	**Viscera Oil**	**Liver Oil**	**Viscera Oil**	**Liver Oil**
0	3.2 ± 0.9 ^abA^	0.8 ± 0.6 ^adB^	1.7 ± 0.3 ^abA^	0.8 ± 0.1 ^aB^
1	4.2 ± 0.4 ^aA^	2.5 ± 0.5 ^bB^	1.1 ± 0.2 ^aA^	1.6 ± 0.0 ^bB^
2	2.1 ± 0.4 ^bcA^	1.5 ± 0.1 ^cdA^	1.4 ± 0.1 ^aA^	2.7 ± 0.2 ^cB^
3	1.5 ± 0.5 ^cA^	1.9 ± 0.3 ^bcA^	2.4 ± 0.1 ^bA^	4.9 ± 0.1 ^dB^
4	12.0 ± 1.0 ^dA^	1.2 ± 0.2 ^dB^	4.6 ± 0.2 ^cA^	5.4 ± 0.1 ^eB^
6	8.6 ± 0.4 ^eA^	0.1 ± 0.0 ^aB^	8.5 ± 0.3 ^dA^	4.5 ± 0.1 ^dB^
**TOTOX**			**TBARS (μmol/g lipid)**	
Day	Viscera oil	Liver oil	Viscera oil	Liver oil
0	8.1	2.3	0.2 ± 0.1 ^aA^	0.1 ± 0.0 ^aA^
1	9.5	6.6	0.3 ± 0.0 ^abA^	0.3 ± 0.0 ^bA^
2	5.6	5.8	0.2 ± 0.2 ^abA^	0.2 ± 0.0 ^bcA^
3	5.4	8.7	0.5 ± 0.5 ^abA^	0.1 ± 0.0 ^acB^
4	28.6	7.8	0.8 ± 0.1 ^bA^	0.2 ± 0.0 ^bB^
6	25.8	4.7	0.6 ± 0.2 ^abA^	0.5 ± 0.0 ^dA^

Different letters within each column (^a–e^) and within the row (^A–B^) of PV, AV and TBARS indicates a significant difference (*p* < 0.05) between samples.

## Data Availability

Data is contained within the article.
